# Non-Participation during Azithromycin Mass Treatment for Trachoma in The Gambia: Heterogeneity and Risk Factors

**DOI:** 10.1371/journal.pntd.0003098

**Published:** 2014-08-28

**Authors:** Tansy Edwards, Elizabeth Allen, Emma M. Harding-Esch, John Hart, Sarah E. Burr, Martin J. Holland, Ansumana Sillah, Sheila K. West, David Mabey, Robin Bailey

**Affiliations:** 1 MRC Tropical Epidemiology Group, London School of Hygiene and Tropical Medicine, London, United Kingdom; 2 Department of Medical Statistics, London School of Hygiene and Tropical Medicine, London, United Kingdom; 3 Department of Clinical Research, London School of Hygiene and Tropical Medicine, London, United Kingdom; 4 Disease Control and Elimination Theme, Medical Research Council Unit (MRC), Fajara, Banjul, The Gambia; 5 National Eye Health Programme, Ministry of Health and Social Welfare, Kanifing, The Gambia; 6 Dana Center for Preventive Ophthalmology, Johns Hopkins University, Baltimore, Maryland, United States of America; University of California San Diego School of Medicine, United States of America

## Abstract

**Background:**

There is concern that untreated individuals in mass drug administration (MDA) programs for neglected tropical diseases can reduce the impact of elimination efforts by maintaining a source of transmission and re-infection.

**Methodology/Principal Findings:**

Treatment receipt was recorded against the community census during three MDAs with azithromycin for trachoma in The Gambia, a hypo-endemic setting. Predictors of non-participation were investigated in 1–9 year olds using random effects logistic regression of cross-sectional data for each MDA. Two types of non-participators were identified: present during MDA but not treated (PNT) and eligible for treatment but absent during MDA (EBA). PNT and EBA children were compared to treated children separately. Multivariable models were developed using baseline data and validated using year one and two data, with *a priori* adjustment for previous treatment status. Analyses included approximately 10000 children at baseline and 5000 children subsequently. There was strong evidence of spatial heterogeneity, and persistent non-participation within households and individuals. By year two, non-participation increased significantly to 10.4% overall from 6.2% at baseline, with more, smaller geographical clusters of non-participating households. Multivariable models suggested household level predictors of non-participation (increased time to water and household head non-participation for both PNT and EBA; increased household size for PNT status only; non-inclusion in a previous trachoma examination survey and younger age for EBA only). Enhanced coverage efforts did not decrease non-participation. Few infected children were detected at year three and only one infected child was EBA previously. Infected children were in communities close to untreated endemic areas with higher rates of EBA non-participation during MDA.

**Conclusions/Significance:**

In hypo-endemic settings, with good coverage and no association between non-participation and infection, efforts to improve participation during MDA may not be required. Further research could investigate spatial hotspots of infection and non-participation in other low and medium prevalence settings before allocating resources to increase participation.

## Author Summary

As the target year for Global Elimination of Trachoma (GET2020) approaches, the scale up of mass drug administration (MDA) with azithromycin will lead to more endemic areas becoming low prevalence settings. In such areas, identification of those at highest risk of *Chlamydia trachomatis* infection and at highest risk of non-participation during MDA could inform control planning, especially if correlation is present. We investigated non-participation in children aged 1–9 years during three annual MDAs in The Gambia, a low prevalence setting. We found evidence that non-participation is associated with household membership and decision-making, as seen in medium and high prevalence settings in East Africa. In addition, we demonstrate geographical heterogeneity (spatial clustering) of non-participation, persistent non-participation behaviour over time and different non-participator types. Between the first and third MDA, non-participation increased significantly overall from 6.2% to 10.4%, whilst spatial clusters became smaller with non-participation more focused in single households or small groups of households. There was no evidence of association between infection and non-participation. In low prevalence settings with no evidence to suggest non-participation as a risk factor for infection, resources to improve participation may not be required. Spatial hotspot analysis could address this research question in areas with more infection.

## Introduction

Trachoma is a leading cause of preventable blindness in endemic areas [1]. Control is through the SAFE strategy [2], of which a key component is mass drug administration (MDA) with the antibiotic azithromycin. Entire communities are targeted during MDA in order to reach both pre-school and school aged children who form the reservoir of infection for *Chlamydia trachomatis*, the causative bacterial agent for trachoma [3].

There is renewed commitment from the World Health Organization (WHO), donors of funding for disease control and research and also pharmaceutical companies to support efforts to eliminate Neglected Tropical Diseases (NTDs), including trachoma, by 2020 [4]–[6]. The success of MDA for NTDs is thought to depend heavily on adequate population coverage in affected areas and participation amongst those offered treatment [7], [8]. With increasing provision of MDA for trachoma, prevalence is expected to fall so that endemic areas will, over time, become low prevalence settings on a trajectory towards the endgame of elimination [4]. In such settings, MDA participation amongst those at highest risk of infection is important. If spatial clusters, or hotspots, of non-participation occur during MDA and correlate with hotspots of infection, it is possible that reservoirs of infection could remain to facilitate continued transmission [9]. This would in turn increase the time needed to reach elimination goals. Identification of factors associated with persistent non-participation in low prevalence settings could therefore provide important clues about how to minimise non-participation. Determining whether infected individuals are amongst non-participators in previous annual MDAs may also provide information regarding the importance of non-participation in low prevalence areas and the potential need for resources to improve participation.

*C. trachomatis* infection, follicular trachoma (TF) and non-participation with azithromycin MDA have all been found to cluster within communities and also within households [10]–[16]. Limited data on non-participation in trachoma control suggest that non-participation is associated mainly with household level decision-making factors, related to knowledge and awareness of trachoma control and also mode of delivery (for example, perception of community drug distributors). A case-control study in Tanzania found household level risk factors such as guardians of children reporting better health in themselves, increased burden due to poor family health, more children per household and younger guardians [3]. At community level, enhanced effort to increase coverage during implementation of MDA was successful in achieving higher participation rates. Studies in Nigeria and South Sudan identified prior household head knowledge of trachoma control and prior notification of MDA as factors associated with better participation but no association with age or gender [17], [18]. In a cluster randomised trial (CRT) in Ethiopia, women and younger children were more likely to be non-participators [15].

For CRTs evaluating the impact of MDA intervention, non-participation can be problematic as it can reduce power to detect intention-to-treat effects [19] and lead to bias in results if there is systematic or heterogeneous non-participation due to reasons also associated with the outcome [20], [21].

In the Partnership for Rapid Elimination of Trachoma (PRET) CRT in The Gambia [22], [23] which represents a hypo-endemic setting (prevalence of TF of 10–20%[7], [24]), MDA took place over a three year period to evaluate the effectiveness of different frequency and coverage MDA delivery strategies on *C. trachomatis* infection and TF in children aged 0–5 years. The aims of this study are to quantify non-participation amongst children aged 1–9 years during PRET, to identify factors associated with non-participation of different types at child, household and community level, to investigate the presence of heterogeneity of non-participation at household and, or community level and determine if any observed household or community heterogeneity is spatially clustered.

## Methods

### Ethical approval

Approval was obtained from the London School of Hygiene & Tropical Medicine Ethics Committee, and The Gambia Government/Medical Research Council Unit, The Gambia Joint Ethics Committee. Written informed consent was obtained from a parent or guardian prior to examination for all children.

### Study design

In PRET, 48 communities (enumerations areas, or EAs) were randomised in a 2×2 factorial design to MDA delivery strategies [11], [22], [23]. A *frequency* strategy allocation resulted in either three annual MDAs of all community members in 24 communities or MDA at baseline only in the remaining 24 communities. A *coverage* strategy allocation (24 communities per arm) was either standard (one day visit to each community by the treatment team of National Eye Health Program (NEHP) in The Gambia) or enhanced (two visits to each community to achieve higher coverage). At the end of the trial, the overall prevalence of TF was around 3% and of *C. trachomatis*, less than 1%.

**Figure 1:**
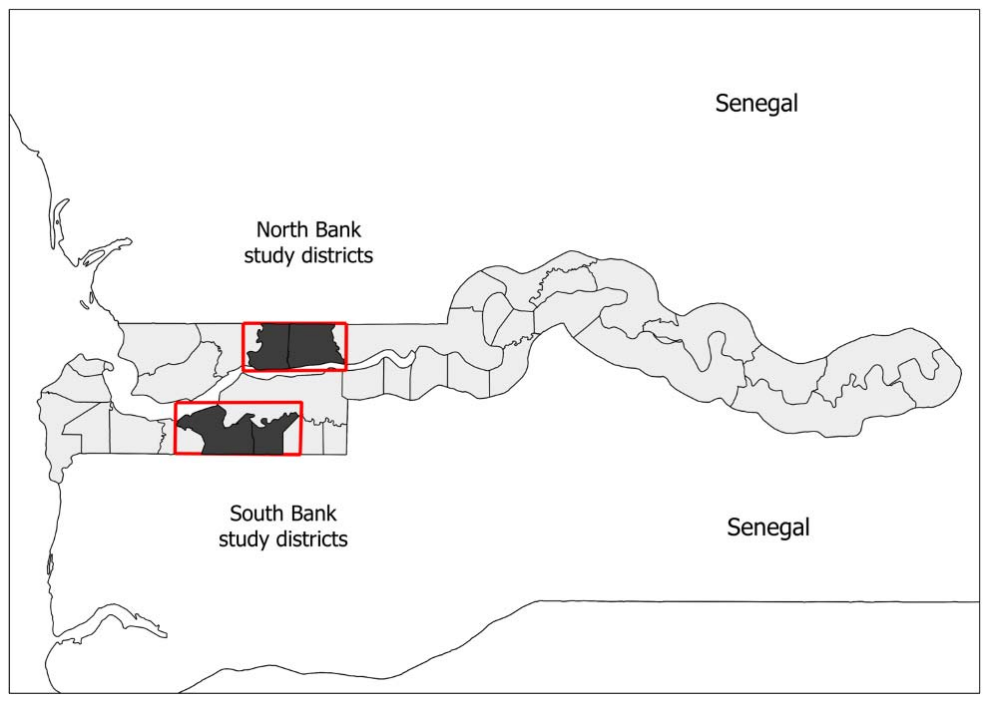
Location of the study districts within The Gambia. Dark grey: study districts, pale grey: remaining districts of The Gambia.

All community members in treated EAs were eligible to receive azithromycin, with the exception of pregnant women and children under six months old who were offered tetracycline ointment if needed. The study took place in two adjacent districts on the northern Bank of the River Gambia and two adjacent districts on the southern Bank (Figure 1) identified for azithromycin MDA. Twelve EAs per district were randomly selected so that only one EA within settlements of more than one EA was chosen. A restricted randomisation of EAs within districts to trial arms was performed by the trial statistician, such that all EA within larger settlements of multiple EA received the same allocation to avoid contamination.

Every six months, between baseline and 36 months inclusive, a complete census was taken. Following this, a random sample of children aged 0–5 years was taken from each community in order to measure trachoma outcomes (the primary outcomes of PRET; presence of TF and *C. trachomatis* infection). Full details of survey methods, sampling strategies and measurement of trachoma outcomes are published elsewhere [11], [22]. MDA took place within approximately one month of the examination rounds. Treatment receipt for each individual was recorded against the census.

### Treatment

A central treatment station was set up in each community during MDAs. Adults aged 14 years or above received 1 g of azithromycin and height was used as a surrogate for weight for children's dosing on the basis of 20 mg/kg [25]. Treatment was administered and directly observed by NEHP treatment teams and the number of tablets or ml of suspension recorded within pre-printed fields included in census forms.

NEHP staff attended the initial training workshop for the PRET trial. Prior to each MDA, treatment team leaders received training about recording treatment status on census forms from the trial coordinator and about dosing from NEHP. Team leaders trained their team. Data review and feedback took place throughout MDAs. Communities were sensitised to MDA by the trial field team before fieldwork started. During the census prior to treatment, the study was again explained to households, and the expected dates for examination and treatment teams' visits were explained. Supervisory field visits were conducted by the NEHP to ensure appropriate distribution. Treatment team members were given per diems to cover food and accommodation for days spent in the field, as a single payment at the end of the fieldwork based on the expected number of days needed.

For each MDA, treatment receipt and eligibility were categorised according to one of the following categories:

- Ineligible: not yet added to the cohort, deceased before MDA, moved elsewhere
- Present not treated (PNT): eligible for treatment as a resident in the census and slept in the household the night before the MDA, but not presenting for treatment
- Treated
- Eligible for treatment but absent (EBA): eligible for treatment as a resident in the census but absent on the treatment day (did not sleep in the household the night before the treatment)
- Eligible for treatment but status unknown (EBU): treatment status not recorded.

### Outcomes and other data

Two binary outcomes were analysed for each MDA; 1) PNT versus treated and 2) EBA versus treated.

EA level variables included coverage allocation, North or South river bank and district, EA type (single settlement, multi-settlement, or segment of a settlement) and population size (small: <600, medium: 600–800, large: >800 individuals). For households, variables included size (small: <11, medium: 11–16, large: >16 individuals), latrine access, time to primary water source, recall of community health education, years of education of household head, a diagnosis of TF for a child aged 0–5 years in the household during the survey immediately prior to the MDA and treatment status of the household head. Child level variables were gender, age, participation in a previous ocular examination survey and treatment status at previous MDAs. Latitude and longitude coordinates were measured for each household.

### Analysis methods

Data were analysed using Stata, version 13 Special Edition [26] and SaTScan [27] and mapped using Quantum GIS [28]. All EAs were treated at baseline and 24 EAs at year one and year two. All available data for children aged 1–9 at the time of each MDA in treated EAs were used to analyse non-participation in this sub-study of the PRET trial. Children with unknown (missing) outcome data were excluded.

The number (%) of children treated, PNT or EBA was summarised overall and by characteristics of interest for each MDA, treating each as a cross-sectional survey (Table S1). Using random effects logistic regression, multivariable models were developed for both outcomes using the baseline data. EA level random intercepts were included in all models and household level random intercepts for EBA versus treated comparisons. PNT children were too few to include a household level random effect. Factors associated with the outcome by a likelihood ratio test (LRT) p-value of <0.1 in univariable analyses were included in a step-wise model building approach to obtain a final multivariable model. Coverage delivery allocation was included in all multivariable models *a priori* since by design the enhanced allocation was intended to increase participation. The same multivariable models were fitted to the year one and two MDA data for validation. Treatment status at previous treatment rounds was added to each of these final models *a priori*. Tests for interaction with coverage allocation were pre-specified if an association between coverage allocation and the outcome was found. Intracluster correlation coefficients (ICCs) with corresponding 95% confidence intervals were obtained from final multivariable models.

Considering the study areas north and south of the River Gambia separately, spatial point patterns were investigated using Kulldorf's scan statistic [29] for each MDA round (baseline, year one and year two), in order to test whether PNT and EBA cases were randomly distributed over space compared to treated children and to identify the location of any significant spatial clusters. Within SatScan software, a circular window is moved systematically throughout the geographic space to identify clusters by centring the window on each household location with a window size of 0% to 50% of the study population, to allow detection of small and large clusters. Clusters containing more than 50% of the population are ignored. A LRT test for a Poisson based model was conducted for each location and size of scanning window to test the hypothesis of an increased rate of non-participator type compared with the distribution outside the window. P-values corresponding to the most likely and secondary clusters are determined using Monte Carlo replications of the dataset. Spatial clusters of PNT and EBA children were added to maps showing the location of children and their treatment status. The locations of infected children at year three are shown on the map for the year two MDA for visual inspection.

## Results

Treatment status was unknown for 403 (3.6%), 88 (1.6%) and 187 (3.0%) eligible children at baseline, year one and year two, respectively. Participation was high overall during each MDA. The overall prevalence of non-participation at baseline was 6.2% (604/9777) with 1.0% (99/9777) of children being PNT and 5.2% (505/9777) of children EBA (Table S1). The distribution of treatment status was similar at year one. Over the three MDAs, the percentage of EBA children appeared to increase and the percentage of PNT children to decrease. By year two, overall non-participation increased to 10.4% (paired t-test of EA summary data p<0.01) due to the increase in EBA children. Reductions in PNT non-participation were not significant.

Of 1626 households eligible for treatment in 24 annually treated communities, one household (0.1%) had PNT children in all three MDAs and 34 (2.1%) had EBA children in all three MDAs. Persistent EBA households were generally larger and within EAs comprised of multiple settlements. The persistent PNT household was further from water, without latrine access and with a household head with no recall of health education or education.

Univariable analyses of baseline data are presented in Table 1. The final multivariable model for being PNT versus treated at baseline included coverage allocation, time to water, household size, household head treatment status and district (Table 2). Children residing in a medium or large household compared to small (p<0.001) and within 15 minutes of primary water source (p<0.001) were less likely to be PNT. A child was more likely to be PNT if the household head was untreated (p<0.001). An association with district was also found (p = 0.002), due to a difference between districts south of the River Gambia. No effect of coverage allocation was found (p = 0.842). A TF diagnosis in the household during the baseline examination round, approximately one month prior to treatment, was associated with lower odds of being PNT in univariable analyses (Table 1) but not after adjustment for other factors in the final model.

**Table 1:** Baseline univariable analysis.

		PNT vs Treated^a^ N = 9272		EBA vs Treated^b^ N = 9678	
Characteristic		OR (95 CI)	LRT p-value^c^	OR (95 CI)	LRT p-value^c^
Coverage	Standard	1		1	
	Enhanced	0.93 (0.25–3.53)	0.916	0.58 (0.34–0.99)	0.051
Bank	South	1		1	
	North	0.18 (0.04–0.85)	0.016	1.28 (0.74–2.22)	0.375
District	South: District 1	1		1	
	South: District 2	9.43 (1.65–54.2)	0.002	0.52 (0.25–1.09)	0.288
	North: District 1	0.33 (0.03–3.34)		0.86 (0.41–1.82)	
	North: District 2	1.62 (0.25–10.7)		1.00 (0.48–2.07)	
EA type	Multiple-SET	1		1	
	Multiple-EA	0.42 (0.10–1.84)	0.289	1.23 (0.66–2.28)	0.793
	Single EA-SET	0.25 (0.03–2.02)		1.15 (0.51–2.59)	
EA size	Small	1		1	
	Medium	2.19 (0.43–11.3)	0.386	0.62 (0.33–1.18)	0.292
	Large	3.23 (0.59–17.7)		0.65 (0.33–1.28)	
HH size	Small	1		1	
	Medium	0.43 (0.26–0.69)	<0.001	0.93 (0.67–1.30)	0.921
	Large	0.22 (0.12–0.40)		0.97 (0.69–1.37)	
Latrine access	No	1		1	
	Yes	0.54 (0.26–1.10)	0.106	1.13 (0.69–1.85)	0.640
Time to water	≥15 mins	1		1	
	<15 mins	0.48 (0.28–0.80)	0.005	0.58 (0.38–0.87)	0.010
Recall of health education	No	1		1	
	Yes	1.25 (0.71–2.23)	0.443	0.70 (0.50–0.98)	0.037
Years of education of head	<1 year	1		1	
	≥1 year	1.31 (0.64–2.68)	0.472	0.91 (0.48–1.71)	0.764
Gender	Male	1		1	
	Female	0.76 (0.50–1.15)	0.188	1.11 (0.90–1.37)	0.322
Age (years)	6–9	1		1	
	3–5	1.00 (0.62–1.61)	0.396	1.02 (0.80–1.30)	<0.001
	1–2	1.39 (0.83–2.31)		1.65 (1.27–2.15)	
TF diagnosis in HH prior to treatment	No	1		1	
	Yes	0.39 (0.15–0.99)	0.025	0.94 (0.61–1.46)	0.786
Previous ocular exam	Yes	1		1	
	No	1.46 (0.92–2.30)	0.101	2.85 (2.17–3.75)	<0.001
Household head treatment status^d^	Treated	1		1	
	PNT	-	<0.001	1.42 (0.47–4.34)	0.001
	EBA	-		2.68 (1.68–4.29)	
	Untreated or EBU	3.85 (2.38–6.22)		-	
	Ineligible or EBU	-		0.85 (0.33–2.17)	

a EA level random effect included in logistic regression model.

b EA and household (HH) random effects included in logistic regression model.

c LRT = likelihood ratio test of **overall** association, comparing models with and without characteristic of interest.

d re-grouping of categories necessary due to zero events in some categories.

**Table 2:** Multivariable models for PNT versus treated children.

		Baseline N = 9272		Year one N = 5131		Year two N = 5479	
Characteristic		OR (95 CI)	LRT p-value^b^	OR (95 CI)	LRT p-value^b^	OR (95 CI)	LRT p-value^b^
Coverage	Standard	1		1		1	
	Enhanced	1.14 (0.32–4.00)	0.842	2.17 (0.16–29.0)	0.557	1.02 (0.08–12.8)	0.988
District	South: District 1	1					
	South: District 2	9.66 (1.72–54.1)	0.002				
	North: District 1	0.42 (0.04–4.14)					
	North: District 2	1.33 (0.21–8.91)					
HH size	Small	1		1		1	
	Medium	0.45 (0.28–0.73)	<0.001	0.94 (0.38–2.33)	0.375	0.81 (0.25–2.63)	0.370
	Large	0.23 (0.13–0.42)		1.78 (0.68–4.64)		0.43 (0.12–1.48)	
Time to water	≥15 mins	1		1		1	
	<15 mins	0.37 (0.22–0.62)	<0.001	7.01 (1.05–47.1)	0.019	0.09 (0.03–0.30)	<0.001
Household head treatment status^a^	Treated	1		1		1	
	PNT	-	<0.001	-	<0.001	-	<0.001
	EBA	-		-		-	
	Ineligible	-		1.99 (0.24–16.2)		-	
	EBU	-		16.0 (4.11–62.6)		-	
	PNT or EBA			36.2 (16.4–80.0)		-	
	Untreated or EBU	3.90 (2.38–6.40)		-		12.4 (4.57–33.6)	
Baseline treatment status^a^	Treated	-		1		1	
	PNT	-		40.2 (4.73–341.8)	0.034	3.43 (0.17–67.8)	0.656
	EBA	-		1.01 (0.13–9.28)		-	
	Ineligible			0.94 (0.32–2.72)		-	
	Eligible-unknown			1.68 (0.45–6.25)		-	
	EBA, ineligible, EBU			-		0.80 (0.25–2.52)	
Year one treatment status^a^	Treated	-				1	
	PNT	-				11.7 (1.27–108.6)	0.032
	EBA	-				6.19 (1.44–26.5)	
	Ineligible or EBU					2.43 (0.67–8.79)	
ICC (EA)		0.43 (0.25–0.63)		0.62 (0.33–0.84)		0.60 (0.26–0.86)	

Models include an EA level random effect.

a re-grouping required due to zero PNT children in some categories.

b LRT = likelihood ratio test of **overall** association, comparing models with and without characteristic of interest.

The same final model was fitted to the year one and year two data, adding previous treatment status. For these follow-up MDAs, the fixed term for district was removed due to zero PNT cases north of the river. Treatment status one year previously was an important predictor of non-participation at both years one and two, with children who were PNT at the previous round being more likely to be PNT again the following year (baseline treatment status at year one MDA: p = 0.034, year one treatment status at year two MDA: p = 0.032, Table 2). Treatment status at baseline was not associated with being PNT at year two (p = 0.656).

The final multivariable model for being EBA versus treated at baseline (Table 3) suggested being EBA was more likely for children who were not included in the baseline examination round (p<0.001), aged 3–5 or 1–2 years compared to 6–9 years (p<0.001), whose household head was also EBA compared to treated, who resided in households further from water (p = 0.018) and possibly for those whose household head could not recall community health education (p = 0.060). Coverage allocation was not associated with being EBA (p = 0.166). Children who were EBA at each previous round were more likely to be EBA at later time points (Table 3). Results also suggest that children who were ineligible at both previous treatment rounds were more likely to be EBA at year two.

**Table 3:** Multivariable models for **EBA** versus treated children.

		Baseline N = 9678		Year one N = 5459		Year two N = 6064	
Characteristic		OR (95 CI)	LRT p-value^b^	OR (95 CI)	LRT p-value^b^	OR (95 CI)	LRT p-value^b^
Coverage	Standard	1		1		1	
	Enhanced	0.62 (0.32–1.24)	0.166	0.53 (0.28–1.17)	0.132	0.77 (0.47–1.26)	0.314
Time to water	≥15 mins	1		1		1	
	<15 mins	0.59 (0.38–0.91)	0.018	2.27 (1.22–4.22)	0.007	0.69 (0.46–1.04)	0.076
Recall of health education	No	1		1		1	
	Yes	0.72 (0.51–1.02)	0.060	0.91 (0.59–1.41)	0.597	1.24 (0.90–1.70)	0.191
Age (years)	6–9	1		1		1	
	3–5	1.82 (1.39–2.39)	<0.001	2.57 (1.79–3.69)	<0.001	1.51 (1.16–1.97)	0.001
	1–2	2.99 (2.23–4.02)		3.62 (2.49–5.27)		1.62 (1.21–2.17)	
Previous ocular exam	Yes	1		1		1	
	No	4.47 (3.29–6.07)	<0.001	2.09 (1.48–2.96)	<0.001	1.35 (1.04–1.75)	0.026
Household head treatment status^a^	Treated	1		1		1	
	PNT	1.49 (0.48–4.63)	0.001	0.53 (0.03–8.33)	<0.001	0.51 (0.08–3.03)	<0.001
	EBA	2.82 (1.73–4.59)		3.43 (1.71–6.88)		4.11 (2.59–6.51)	
	Ineligible	-		3.30 (1.59–6.82)		3.27 (1.03–10.4)	
	EBU	-		3.09 (1.04–9.19)		2.23 (1.23–4.02)	
	Ineligible or EBU	0.95 (0.36–2.53)		-		-	
Baseline treatment status	Treated			1		1	
	PNT			1.88 (0.40–8.75)	<0.001	0.66 (0.12–3.77)	0.011
	EBA			3.97 (2.41–6.52)		1.72 (1.05–2.83)	
	Ineligible			1.03 (0.70–1.50)		1.48 (1.13–1.94)	
	EBU			0.98 (0.37–2.62)		1.81 (0.94–3.52)	
Year one treatment status	Treated					1	
	PNT					0.90 (0.30–2.73)	<0.001
	EBA					7.56 (5.20–11.0)	
	Ineligible					1.54 (1.15–2.06)	
	EBU					0.77 (0.22–2.72)	
ICC (EA)		0.15 (0.09–0.24)		0.08 (0.03–0.18)		0.05 (0.02–0.11)	
ICC (HH)		0.53 (0.46–0.60)		0.51 (0.42–0.59)		0.38 (0.31–0.45)	

Models include EA and household random effects.

a At baseline, re-grouping of household head treatment status was required due to zero EBA in the ineligible household head status group.

b LRT = likelihood ratio test of **overall** association, comparing models with and without characteristic of interest.

In the EBA versus treated comparisons ICCs suggested substantially more variation was present between households within EAs, than between EAs (Table 3). ICCs from PNT models at EA level were closer to the ICCs estimated at household level for EBA children, possibly because between-household variation could not be determined due to the very low prevalence of PNT non-participation.

GPS coordinates were missing for 11 out of 1626 households, excluding 23 children from spatial analyses.

**Figure 2:**
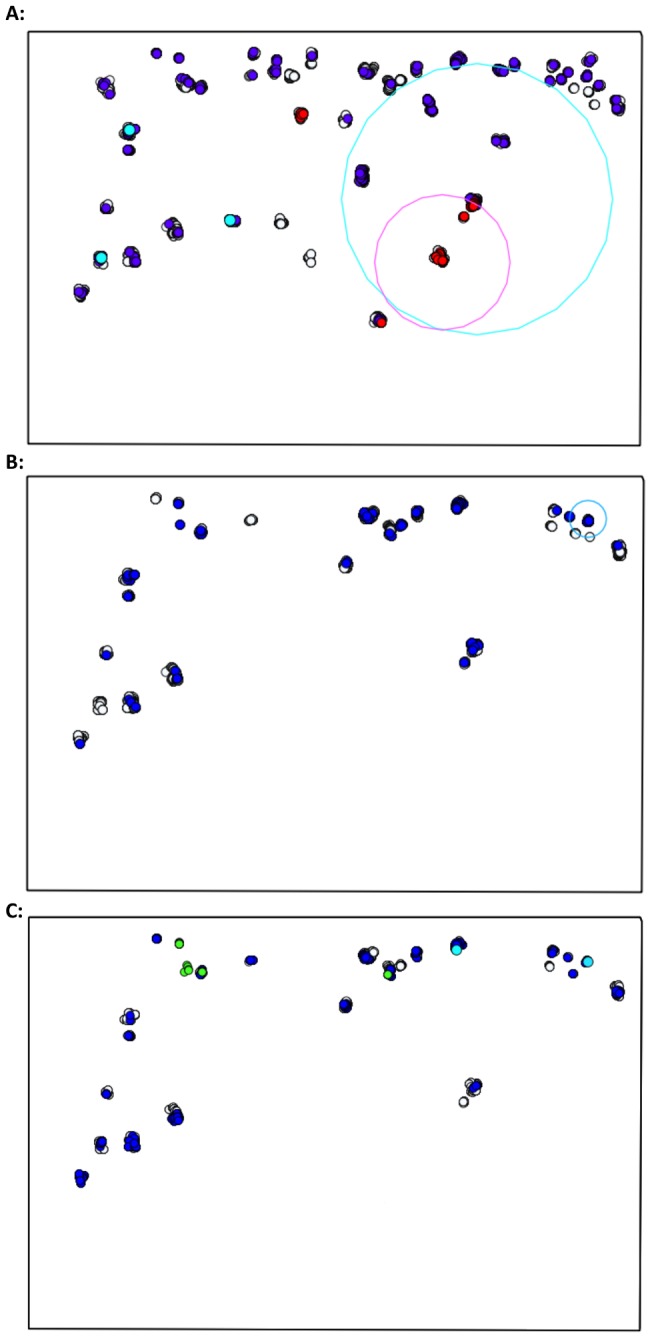
Spatial clusters of PNT and EBA children aged 1–9 years on the North river bank. A: baseline treatment round, B: year one, C: year two. Treated (grey), PNT (red), EBA (blue), PNT cluster (pink), EBA cluster (light blue). No PNT children at year one or year two in study districts north of the river. Location of children aged 0–5 years with *C. trachomatis* infection at year three (green).

**Figure 3:**
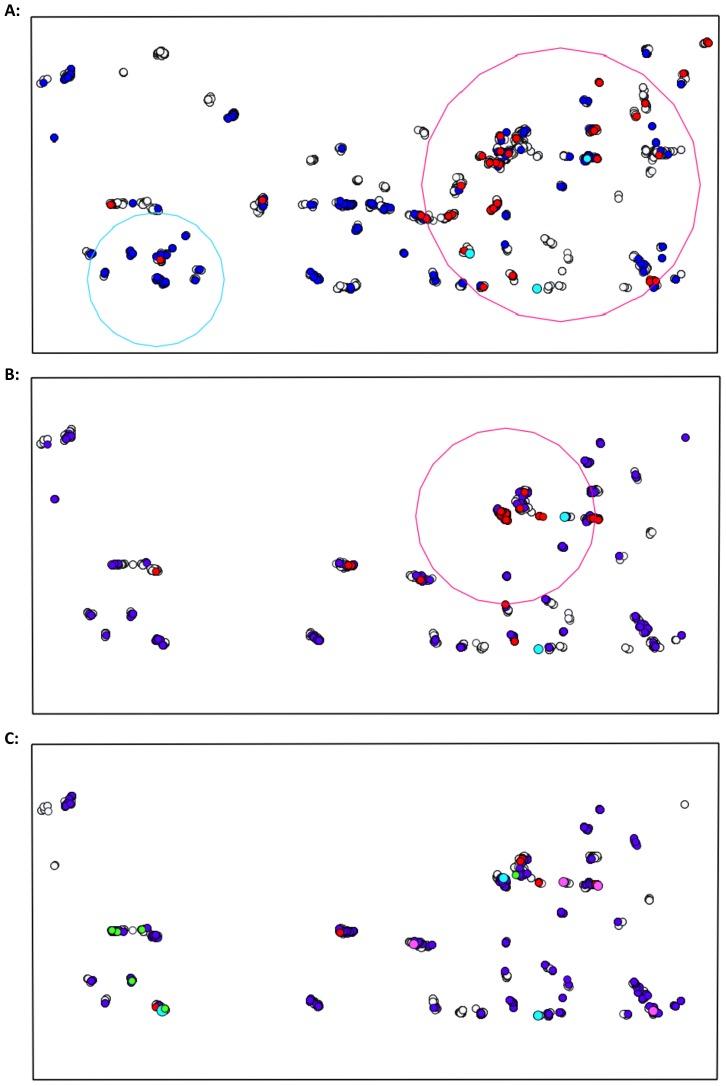
Spatial clusters of PNT and EBA children aged 1–9 years on the South river bank. A: baseline treatment round, B: year one, C: year two. Treated (grey), PNT (red), EBA (blue), PNT cluster (pink), EBA cluster (light blue). Location of children aged 0–5 years with *C. trachomatis* infection at year three (green).

Spatial clusters of PNT and EBA children were detected at baseline in study areas on each side of the river (Table 4). No PNT children were reported in year one or year two in the northern river bank districts. Spatial clusters of PNT and EBA children reduced in size in each subsequent MDA and by year two, clusters included either single households or a small group of adjacent households (Figures 2 and 3).

**Table 4:** Spatial clusters of non-participation.

Type	Clusters	Radius (km)	p-value
Baseline:			
North River Bank			
PNT	1	3.13	<0.001
EBA	1	6.27	<0.001
	2	0.062	0.001
	3	0.048	0.002
	4	0	0.010
South River Bank			
PNT	1	7.43	<0.001
EBA	1	3.64	<0.001
	2	0.054	<0.001
	3	0	0.001
	4	0.22	0.010
Year one:			
North River Bank^a^			
EBA	1	0.85	<0.001
South River Bank			
PNT	1	4.80	<0.001
EBA	1	0	<0.001
	2	0.12	0.001
Year two:			
North River Bank^a^			
EBA	1	0.079	<0.001
	2	0.080	0.027
South River Bank			
PNT	1	0	<0.001
	2	0	0.002
	3	0	0.002
	4	0	0.0002
EBA	1	0.25	<0.001
	2	0.026	0.001
	3	0.35	0.013

a no PNT children at year one or year two in districts north of the River Gambia.

Cases of *C. trachomatis* infection in annually treated communities at year three (n = 14) were found within three kilometres of Senegal in all but one child. In Senegalese districts adjacent to The Gambia, MDA had not yet taken place. Infections were detected amongst children who were ineligible or treated during the three prior MDAs, apart from one child residing on the north side of the river who was persistently EBA during the MDAs. Two cases were located in an EA with households within a year two EBA cluster on the south side of the river (Figures 2 and 3). In the two EAs with households in this spatial cluster, approximately 15% of 1–9 year olds were EBA during the year two MDA.

## Discussion

In this large study of non-participation during azithromycin MDA from a low prevalence trachoma setting, we demonstrate further evidence of heterogeneity of non-participation in children aged 1–9 years, particularly at household level, in line with studies in higher prevalence settings. We also observed persistent non-participation over time in annual MDAs, as seen elsewhere in a CRT setting [3]. Geographical clustering of non-participation represents a new finding and we found two different types of non-participators. We found circumstantial rather than statistical evidence of an association between infection and non-participation during a previous MDA, however, the overall prevalence of infection and TF in 0–5 year olds at the end of PRET was below a level requiring any SAFE interventions. Detection of infection in communities close to untreated areas [22], relatively high EBA rates in those communities during the previous MDA and literature from The Gambia and elsewhere linking travel with re-infection [30], [31] together, suggest the observed infections could have resulted from exposure to untreated persons. Travel plans could have been set prior to notification of MDA timing and therefore could have been unrelated to intentional non-participation, although intentional decision making to miss treatment is a possibility.

Household level variables were associated with greater likelihood of being PNT and EBA. Household head non-participation and their type of non-participation predicted PNT and EBA status in children, implying household decision making with respect to MDA participation behaviour. The finding that children in households further from their primary water source were more likely to be PNT or EBA is probably indicative of some other unmeasured risk factor, for example, marginalisation within the community due to either household head or community leader choice, or a mixture of the two. Non-participation during MDA subsequent to participation in a previous MDA has been found to be associated with possible markers of marginalisation in another CRT [32]. Active trachoma has been found to be associated with lower socio-economic status (SES) and isolation of households from the community [33] so access to, or participation in, trachoma control activities could also be affected by these unmeasured factors. Smaller household size was important for predicting PNT status but not EBA, compared to treated children, which could represent some effect of lower SES. Recent migration into the community could also mean less access to community decision making and activities. Participation in a previous TF examination survey could be indicative of increased awareness and acceptance of control activities in annually treated communities, however, a proxy effect cannot be concluded in case of potential bias introduced by households more willing to take part in all control and assessment activities. Results from the Gambian setting suggest that enhanced efforts to increase coverage of mass treatment programs, by means of an extra treatment team visit to the community do not improve participation, in contrast to the PRET trial conducted in Tanzania [3].

Studies of MDA participation in Africa for onchocerciasis and lymphatic filariasis, other NTDs for which control is through mass community-wide treatment, have also linked non-participation to household level decision making factors, for example, a perception of low disease risk or lack of family or household support [34]–[36]. The Gambia has relatively high childhood immunisation coverage [37], elimination of trachoma by 2020 is attainable [24] and non-participation was higher in the districts south of the river where the prevalence of TF was consistently lower during PRET [22]. It is perhaps plausible therefore that a household level decision based on a perceived lack of need for treatment could apply in this low prevalence setting, although we do not have data from each community to assess this. Reasons for being EBA in this setting could be logistic and independent of participation choices, for example, population movement and travel where children are sent away for weaning which is common practice in The Gambia, or farming related activities. PNT and EBA comparisons to treated children were performed separately as it was hypothesised that there may be differences in reasons for non-participation that may or may not be related to refusal of treatment or a perceived lack of need for treatment. The data do suggest some differences between PNT and EBA children but further information is unavailable to determine if and why there was an active decision to refuse treatment.

Due to the very low prevalence of TF and infection in both MDA frequency arms (annual and baseline only MDA) throughout the original trial, it is unlikely that the heterogeneous non-participation observed here had an additional negative effect on power to detect differences between arms in intention-to-treat analyses in the PRET trial. It is also unlikely that heterogeneous non-participation introduced bias in comparative analyses given the low prevalence of TF and infection. We found a geographical effect on non-participation and on trachoma outcomes [22]. Infections did occur in one part of the study area with notable EBA non-participation at the previous MDA, however, even if all PNT and EBA children at the year two MDA had been found to have infection and TF, the overall prevalence of each outcome at year three would have been less than 5% and thus still below MDA continuation thresholds for TF. Therefore, for the Gambian national trachoma control program, efforts and resources to address non-participation are not required.

For national control programs in low and medium prevalence settings, heterogeneous non-participation linked to increased risk of infection could present challenges for elimination efforts. Links between infection and non-participation in prior MDA rounds could undermine MDA where corresponding prevalence levels for TF meet criteria for continued MDA at the time of impact assessment. Identification of hotspots of infection and non-participation, along with modifiable risk factors for non-participation could take place during impact assessment following repeated MDA. The results could then aid control program managers working towards elimination goals in low and medium prevalence settings, by enabling them to target delivery resources for continued MDA and to improve coverage in areas with a greater threat of continued transmission.

## Supporting Information

Checklist S1 STROBE checklist.
(DOC)

Table S1 Treatment status amongst children aged 1–9 years eligible for treatment at each time point. PNT = Present not treated, EBA = Eligible but absent, EBU = eligible but unknown treatment status.
(DOCX)

## References

[1] Mabey DCW, Solomon AW, Foster A (2003) Trachoma. The Lancet 362: 223–229. 10.1016/S0140-6736(03)13914-1 12885486

[2] Solomon AW (2006) Trachoma control: a guide for programme managers: World Health Organization.

[3] Ssemanda EN, Levens J, Mkocha H, Munoz B, West SK (2012) Azithromycin mass treatment for trachoma control: risk factors for non-participation of children in two treatment rounds. PLoS Negl Trop Dis 6: e1576. 22448296 10.1371/journal.pntd.0001576 PMC3308937

[4] World Health Organization (2012) Accelerating work to overcome the global impact of neglected tropical diseases: A roadmap for implementation.

[5] Uniting to combat NTDs, Ending the Neglect and Reaching 2020 Goals (2012) London Declaration on Neglected Tropical Diseases. http://www.unitingtocombatntds.org/downloads/press/london_declaration_on_ntds.pdf.

[6] World Health Organization (2011) Report of the Fifteenth Meeting of the WHO Alliance for the Elimination of Blinding Trachoma by 2020. Geneva.

[7] Liu F, Porco TC, Mkocha HA, Munoz B, Ray KJ, et al (2014) The efficacy of oral azithromycin in clearing ocular chlamydia: mathematical modeling from a community-randomized trachoma trial. Epidemics 6: 10–7 10.1016/j.epidem.2013.1012.1001. Epub 2014 Jan 1018. 24593917 10.1016/j.epidem.2013.12.001 PMC4420489

[8] Anderson R, Truscott J, Hollingsworth TD (2014) The coverage and frequency of mass drug administration required to eliminate persistent transmission of soil-transmitted helminths. Philos Trans R Soc Lond B Biol Sci 369: 20130435 doi:20130410.20131098/rstb.20132013.20130435. Print 20132014 24821921 10.1098/rstb.2013.0435 PMC4024228

[9] Broman AT, Shum K, Munoz B, Duncan DD, West SK (2006) Spatial clustering of ocular chlamydial infection over time following treatment, among households in a village in Tanzania. Investigative ophthalmology & visual science 47: 99–104. 16384950 10.1167/iovs.05-0326

[10] Edwards T, Harding-Esch EM, Hailu G, Andreason A, Mabey DC, et al (2008) Risk factors for active trachoma and Chlamydia trachomatis infection in rural Ethiopia after mass treatment with azithromycin. Trop Med Int Health 13: 556–565. 18282237 10.1111/j.1365-3156.2008.02034.x

[11] Harding-Esch EM, Edwards T, Mkocha H, Munoz B, Holland MJ, et al (2010) Trachoma Prevalence and Associated Risk Factors in The Gambia and Tanzania: Baseline Results of a Cluster Randomised Controlled Trial. PLoS neglected tropical diseases 4: e861. 21072224 10.1371/journal.pntd.0000861 PMC2970530

[12] Hägi M, Schémann JF, Mauny F, Momo G, Sacko D, et al (2010) Active trachoma among children in Mali: Clustering and environmental risk factors. PLoS neglected tropical diseases 4: e583. 20087414 10.1371/journal.pntd.0000583 PMC2799671

[13] Blake IM, Burton MJ, Bailey RL, Solomon AW, West S, et al (2009) Estimating household and community transmission of ocular Chlamydia trachomatis. PLoS Negl Trop Dis 3: e401. 19333364 10.1371/journal.pntd.0000401 PMC2655714

[14] Edwards T, Smith J, Sturrock HJ, Kur LW, Sabasio A, et al (2012) Prevalence of trachoma in unity state, South Sudan: results from a large-scale population-based survey and potential implications for further surveys. PLoS Negl Trop Dis 6: e1585. 22506082 10.1371/journal.pntd.0001585 PMC3323519

[15] Keenan JD, Ayele B, Gebre T, Zerihun M, Zhou Z, et al (2011) Childhood mortality in a cohort treated with mass azithromycin for trachoma. Clinical infectious diseases 52: 883. 21427395 10.1093/cid/cir069 PMC3106233

[16] Ssemanda EN, Munoz B, Harding-Esch EM, Edwards T, Mkocha H, et al (2010) Mass treatment with azithromycin for trachoma control: participation clusters in households. PLoS Negl Trop Dis 4: e838. 20957196 10.1371/journal.pntd.0000838 PMC2950137

[17] Cromwell EA, King JD, McPherson S, Jip FN, Patterson AE, et al (2013) Monitoring of mass distribution interventions for trachoma in Plateau State, Nigeria. PLoS Negl Trop Dis 7: e1995. 23326617 10.1371/journal.pntd.0001995 PMC3542118

[18] Cromwell EA, Ngondi J, Gatpan G, Becknell S, Kur L, et al (2009) Estimation of population coverage for antibiotic distribution for trachoma control: a comparison of methods. International health 1: 182–189. 24036565 10.1016/j.inhe.2009.09.002

[19] Jo B, Asparouhov T, Muthén BO (2008) Intention-to-treat analysis in cluster randomized trials with noncompliance. Statistics in medicine 27: 5565–5577. 18623608 10.1002/sim.3370 PMC2907896

[20] Sommer A, Zeger SL (1991) On estimating efficacy from clinical trials. Statistics in medicine 10: 45–52. 2006355 10.1002/sim.4780100110

[21] Jo B, Asparouhov T, Muthén BO, Ialongo NS, Brown CH (2008) Cluster randomized trials with treatment noncompliance. Psychological methods 13: 1. 18331150 10.1037/1082-989X.13.1.1 PMC2917590

[22] Harding-Esch EM, Sillah A, Edwards T, Burr SE, Hart JD, et al (2013) Mass treatment with azithromycin for trachoma: when is one round enough? Results from the PRET Trial in the Gambia. PLoS Negl Trop Dis 7: e2115. 23785525 10.1371/journal.pntd.0002115 PMC3681669

[23] Stare D, Harding-Esch E, Munoz B, Bailey R, Mabey D, et al (2011) Design and baseline data of a randomized trial to evaluate coverage and frequency of mass treatment with azithromycin: the Partnership for Rapid Elimination of Trachoma (PRET) in Tanzania and The Gambia. Ophthalmic Epidemiology 18: 20–29. 21275593 10.3109/09286586.2010.545500

[24] Harding-Esch EM, Edwards T, Sillah A, Sarr I, Roberts CH, et al (2009) Active trachoma and ocular Chlamydia trachomatis infection in two Gambian regions: on course for elimination by 2020? PLoS neglected tropical diseases 3: e573. 20027217 10.1371/journal.pntd.0000573 PMC2791206

[25] Muñoz B, Solomon AW, Zingeser J, Barwick R, Burton M, et al (2003) Antibiotic dosage in trachoma control programs: height as a surrogate for weight in children. Investigative ophthalmology & visual science 44: 1464–1469. 12657580 10.1167/iovs.02-0234 PMC6858275

[26] StataCorp (2013) Stata Statistical Software: Release 13. College Station, Texas.

[27] Kulldorff M (2010) SaTScan™ User Guide, version 9.0.

[28] QGIS Development Team (2013) QGIS Geographic Information System. Open Source Geospatial Foundation Project. http://qgis.osgeo.org.

[29] Pullan RL, Sturrock HJ, Soares Magalhaes RJ, Clements AC, Brooker SJ (2012) Spatial parasite ecology and epidemiology: a review of methods and applications. Parasitology 139: 1870–1887. 23036435 10.1017/S0031182012000698 PMC3526959

[30] Burton MJ, Holland MJ, Makalo P, Aryee EA, Alexander ND, et al (2005) Re-emergence of Chlamydia trachomatis infection after mass antibiotic treatment of a trachoma-endemic Gambian community: a longitudinal study. Lancet 365: 1321–1328. 15823382 10.1016/S0140-6736(05)61029-X

[31] Shah NA, House J, Lakew T, Alemayehu W, Halfpenny C, et al (2010) Travel and implications for the elimination of trachoma in ethiopia. Ophthalmic Epidemiol 17: 113–117. 20302432 10.3109/09286581003624921

[32] Ssemanda EN, Mkocha H, Levens J, Munoz B, West SK (2013) Community mass treatment with azithromycin for trachoma: Factors associated with change in participation of children from the first to the second round. Clinical Epidemiology and Global Health DOI:10.1016/j.cegh.2013.06.001 [epub ahead of print]. 10.1016/j.cegh.2013.06.001 PMC4599782 26462290

[33] Montgomery MA, Desai MM, Groce NE, Elimelech M (2011) Relationship between distance to social gathering facilities and risk of trachoma for households in rural Tanzanian communities. Social science & medicine (1982) 73: 1–5. 21641706 10.1016/j.socscimed.2011.05.003

[34] Nuwaha F, Okware J, Ndyomugyenyi R (2005) Predictors of compliance with community-directed ivermectin treatment in Uganda: quantitative results. Trop Med Int Health 10: 659–667. 15960704 10.1111/j.1365-3156.2005.01436.x

[35] Yirga D, Deribe K, Woldemichael K, Wondafrash M, Kassahun W (2010) Factors associated with compliance with community directed treatment with ivermectin for onchocerciasis control in Southwestern Ethiopia. Parasites & vectors 3: 48. 20525182 10.1186/1756-3305-3-48 PMC2896929

[36] Njomo D, Amuyunzu-Nyamongo M, Mukoko D, Magambo J, Njenga S (2012) Socioeconomic factors associated with compliance with mass drug administration for lymphatic filariasis elimination in Kenya: Descriptive study results. Annals of Tropical Medicine and Public Health 5: 103.

[37] World Health Organization (2013) WHO vaccine-preventable diseases: monitoring system. 2013 global summary. http://apps.who.int/immunization_monitoring/globalsummary/coverages?c=GMB.

