# Supplementary material for: Non-Participation during Azithromycin Mass Treatment for Trachoma in The Gambia: Heterogeneity and Risk Factors
Source: PLoS Negl Trop Dis. 2014 Aug 28;8(8):e3098. doi: 10.1371/journal.pntd.0003098 (PMC4148234; doi:10.1371/journal.pntd.0003098)
Supplement: Table S1 — Treatment status amongst children aged 1–9 years eligible for treatment at each time point. PNT = Present not treated, EBA = Eligible but absent, EBU = eligible but unknown treatment status. (DOCX) [file pntd.0003098.s002.docx]

Table S1. Treatment status amongst children aged 1-9 years eligible for treatment at each time point

|  | | Baseline Treatment Status N = 9777 | | | | Year one N = 5504 | | | | Year two N = 6086 | | | |
| --- | --- | --- | --- | --- | --- | --- | --- | --- | --- | --- | --- | --- | --- |
| Characteristic | | N | Treated | PNT | EBA | N | Treated | PNT | EBA | N | Treated | PNT | EBA |
| Total | | 9777 | 9178 (93.8) | 99 (1.0) | 505 (5.2) | 5504 | 5086 (92.4) | 45 (0.8) | 373 (6.8) | 6086 | 5457 (89.6) | 22 (0.4) | 607 (10.0) |
| Coverage | Standard | 4793 | 4436 (92.6) | 48 (1.0) | 309 (6.4) | 2598 | 2379 (91.6) | 23 (0.9) | 196 (7.5) | 2928 | 2590 (88.5) | 9 (0.3) | 329 (11.2) |
|  | Enhanced | 4984 | 4737 (95.1) | 51 (1.0) | 196 (3.9) | 2914 | 2715 (93.2) | 22 (0.8) | 177 (6.1) | 3162 | 2871 (90.8) | 13 (0.4) | 278 (8.8) |
| Bank | South | 4993 | 4712 (94.4) | 52 (1.0) | 229 (4.6) | 2705 | 2414 (89.2) | 45 (1.7) | 246 (9.1) | 3073 | 2680 (87.2) | 22 (0.7) | 371 (12.1) |
|  | North | 4784 | 4461 (93.2) | 47 (1.0) | 276 (5.8) | 2799 | 2672 (95.5) | 0 (0) | 127 (4.5) | 3013 | 2777 (92.2) | 0 (0) | 236 (7.8) |
| District | South: District 1 | 2111 | 1981 (93.8) | 6 (0.3) | 124 (5.9) | 1092 | 1007 (92.2) | 5 (0.5) | 80 (7.3) | 1259 | 1107 (87.9) | 6 (0.5) | 146 (11.6) |
|  | South: District 2 | 2882 | 2731 (94.8) | 46 (1.6) | 105 (3.6) | 1613 | 1407 (87.2) | 40 (2.5) | 166 (10.3) | 1814 | 1573 (86.7) | 16 (0.9) | 225 (12.4) |
|  | North: District 1 | 2099 | 1979 (94.3) | 4 (0.2) | 116 (5.5) | 1190 | 1151 (96.7) | 0 (0) | 39 (3.3) | 1224 | 1130 (92.3) | 0 (0) | 94 (7.7) |
|  | North: District 2 | 2685 | 2482 (92.4) | 43 (1.6) | 160 (6.0) | 1609 | 1521 (94.5) | 0 (0) | 88 (5.5) | 1789 | 1647 (92.1) | 0 (0) | 142 (7.9) |
| EA type | Multiple-SET | 6036 | 5647 (93.6) | 83 (1.4) | 306 (5.1) | 3337 | 3045 (91.3) | 17 (0.5) | 275 (8.2) | 3703 | 3339 (90.2) | 9 (0.2) | 355 (9.6) |
|  | Multiple-EA | 2402 | 2263 (94.2) | 12 (0.5) | 127 (5.3) | 1611 | 1515 (94.0) | 25 (1.6) | 71 (4.4) | 1754 | 1544 (88.0) | 9 (0.5) | 201 (11.5) |
|  | Single EA-SET | 1339 | 1263 (94.3) | 4 (0.3) | 72 (5.4) | 556 | 526 (94.6) | 3 (0.5) | 27 (4.8) | 629 | 574 (91.3) | 4 (0.6) | 51 (8.1) |
| EA size | Small | 2341 | 2188 (93.5) | 8 (0.3) | 145 (6.2) | 1757 | 1638 (93.2) | 2 (0.1) | 117 (6.6) | 786 | 704 (89.6) | 0 (0) | 82 (10.4) |
|  | Medium | 3426 | 3241 (94.6) | 34 (1.0) | 151 (4.4) | 1009 | 963 (95.4) | 6 (0.6) | 40 (4.0) | 1658 | 1476 (89.0) | 8 (0.5) | 174 (10.5) |
|  | Large | 4010 | 3744 (93.4) | 57 (1.4) | 209 (5.2) | 2738 | 2485 (90.8) | 37 (1.3) | 216 (7.9) | 3642 | 3277 (90.0) | 14 (0.4) | 351 (9.6) |
| HH size | Small | 3200 | 2981 (93.1) | 53 (1.7) | 166 (5.2) | 1609 | 1491 (92.7) | 12 (0.7) | 106 (6.6) | 1259 | 1144 (90.8) | 6 (0.5) | 109 (8.7) |
|  | Medium | 3118 | 2936 (94.2) | 29 (0.9) | 153 (4.9) | 1828 | 1703 (93.2) | 18 (1.0) | 107 (5.9) | 1816 | 1640 (90.3) | 9 (0.5) | 167 (9.2) |
|  | Large | 3459 | 3256 (94.1) | 17 (0.5) | 186 (5.4) | 2067 | 1892 (91.5) | 15 (0.7) | 160 (7.7) | 3011 | 2673 (88.8) | 7 (0.2) | 331 (11.0) |
| Latrine access | No | 900 | 847 (94.1) | 11 (1.2) | 42 (4.7) | 605 | 555 (91.7) | 7 (1.2) | 43 (7.1) | 714 | 638 (89.3) | 4 (0.6) | 72 (10.1) |
|  | Yes | 8877 | 8326 (93.8) | 88 (1.0) | 463 (5.2) | 4899 | 4531 (92.5) | 38 (0.8) | 330 (6.7) | 5372 | 4819 (89.7) | 18 (0.3) | 535 (10.0) |
| Time to water | **≥** 15 mins | 1497 | 1350 (90.2) | 39 (2.6) | 108 (7.2) | 757 | 718 (94.9) | 3 (0.4) | 36 (4.7) | 835 | 729 (87.3) | 11 (1.3) | 95 (11.4) |
|  | **<** 15 mins | 8280 | 7823 (94.5) | 60 (0.7) | 397 (4.8) | 4747 | 4368 (92.0) | 42 (0.9) | 337 (7.1) | 5251 | 4728 (90.0) | 11 (0.2) | 512 (9.8) |
| Recall of health education | No | 6612 | 6191 (93.6) | 54 (0.8) | 367 (5.6) | 3821 | 3514 (92.0) | 26 (0.7) | 281 (7.3) | 4266 | 3831 (89.8) | 20 (0.5) | 415 (9.7) |
|  | Yes | 3165 | 2982 (94.2) | 45 (1.4) | 138 (4.4) | 1683 | 1572 (93.4) | 19 (1.1) | 92 (5.5) | 1820 | 1626 (89.3) | 2 (0.1) | 192 (10.6) |
| Years of education of HH head | <1 year | 9204 | 8632 (93.8) | 89 (1.0) | 483 (5.2) | 5169 | 4789 (92.6) | 39 (0.8) | 341 (6.6) | 5728 | 5152 (89.9) | 15 (0.3) | 561 (9.8) |
|  | ≥1 year | 573 | 541 (94.4) | 10 (1.8) | 22 (3.8) | 335 | 297 (88.6) | 6 (1.8) | 32 (9.6) | 358 | 305 (85.2) | 7 (2.0) | 46 (12.8) |
| Gender | Male | 5063 | 4752 (93.9) | 58 (1.1) | 253 (5.0) | 2887 | 2663 (92.2) | 23 (0.8) | 201 (7.0) | 3134 | 2835 (90.4) | 15 (0.5) | 284 (9.1) |
|  | Female | 4714 | 4421 (93.8) | 41 (0.9) | 252 (5.3) | 2617 | 2423 (92.6) | 22 (0.8) | 172 (6.6) | 2952 | 2622 (88.9) | 7 (0.2) | 323 (10.9) |
| Age (years) | 6-9 | 3998 | 3775 (94.4) | 38 (1.0) | 185 (4.6) | 2274 | 2153 (94.6) | 17 (0.8) | 104 (4.6) | 2531 | 2320 (91.7) | 9 (0.4) | 202 (8.0) |
|  | 3-5 | 3591 | 3379 (94.1) | 34 (1.0) | 178 (5.0) | 1888 | 1735 (91.9) | 15 (0.8) | 138 (7.3) | 2019 | 1796 (89.0) | 7 (0.4) | 216 (10.7) |
|  | 1-2 | 2188 | 2019 (92.3) | 27 (1.2) | 142 (6.5) | 1342 | 1198 (89.2) | 13 (1.0) | 131 (9.8) | 1540 | 1345 (87.3) | 6 (0.4) | 189 (12.3) |
| Baseline treatment status | Treated | - | - | - | - | 4340 | 4059 (93.5) | 30 (0.7) | 251 (5.8) | 3873 | 3552 (91.7) | 13 (0.3) | 308 (8.0) |
|  | PNT | - | - | - | - | 35 | 29 (82.8) | 3 (8.6) | 3 (8.6) | 35 | 31 (88.6) | 2 (5.7) | 2 (5.7) |
|  | EBA | - | - | - | - | 244 | 195 (79.9) | 1 (0.4) | 48 (19.7) | 236 | 198 (83.9) | 0 (0) | 38 (16.1) |
|  | Ineligible | - | - | - | - | 758 | 690 (91.0) | 5 (0.7) | 63 (8.3) | 1832 | 1590 (86.8) | 7 (0.4) | 235 (12.8) |
|  | EBU | - | - | - | - | 127 | 113 (89.0) | 6 (4.7) | 8 (6.3) | 114 | 90 (79.0) | 0 (0) | 24 (21.1) |
| Year one treatment status | Treated | - | - | - | - | - | - | - | - | 4683 | 4320 (92.3) | 11 (0.2) | 352 (7.5) |
|  | PNT | - | - | - | - | - | - | - | - | 50 | 41 (82.0) | 3 (6.0) | 6 (12.0) |
|  | EBA | - | - | - | - | - | - | - | - | 283 | 188 (66.4) | 3 (1.1) | 92 (32.5) |
|  | Ineligible | - | - | - | - | - | - | - | - | 1028 | 871 (84.7) | 5 (0.5) | 152 (14.8) |
|  | EBU | - | - | - | - | - | - | - | - | 46 | 41 (89.1) | 0 (0) | 5 (10.9) |
| TF diagnosis in HH prior to treatment | No | 8435 | 7897 (93.6) | 94 (1.1) | 444 (5.3) | 5173 | 4768 (92.1) | 45 (0.9) | 360 (7.0) | 5717 | 5114 (89.5) | 22 (0.4) | 581 (10.2) |
|  | Yes | 1342 | 1276 (95.1) | 5 (0.4) | 61 (4.6) | 331 | 318 (96.1) | 0 (0) | 13 (3.9) | 373 | 347 (93.0) | 0 (0) | 26 (7.0) |
| Previous ocular exam | Yes | 4827 | 4594 (95.2) | 33 (0.7) | 200 (4.1) | 3561 | 3331 (93.5) | 23 (0.7) | 207 (5.8) | 4386 | 3967 (90.5) | 16 (0.4) | 403 (9.2) |
|  | No | 4950 | 4579 (92.5) | 66 (1.3) | 305 (6.2) | 1943 | 1755 (90.4) | 22 (1.1) | 166 (8.5) | 1704 | 1494 (87.7) | 6 (0.4) | 204 (12.0) |
| Household head treatment status | Treated | 8678 | 8189 (94.4) | 70 (0.8) | 419 (4.8) | 4815 | 4529 (94.0) | 13 (0.3) | 273 (5.7) | 5294 | 4825 (91.1) | 7 (0.1) | 462 (8.7) |
|  | PNT | 161 | 124 (77.0) | 28 (17.4) | 9 (5.6) | 47 | 21 (44.7) | 25 (53.2) | 1 (2.1) | 48 | 39 (81.2) | 7 (14.6) | 2 (4.2) |
|  | EBA | 626 | 560 (89.5) | 1 (0.2) | 65 (10.4) | 262 | 218 (83.2) | 2 (0.8) | 42 (16.0) | 417 | 324 (77.7) | 0 (0) | 93 (22.3) |
|  | Ineligible | 33 | 33 (100) | 0 (0) | 0 (0) | 255 | 211 (82.7) | 1 (0.4) | 43 (16.9) | 65 | 48 (73.9) | 0 (0) | 17 (26.1) |
|  | EBU | 279 | 267 (95.7) | 0 (0) | 12 (4.3) | 125 | 107 (85.6) | 4 (3.2) | 14 (11.2) | 262 | 221 (84.4) | 8 (3.1) | 33 (12.6) |
